# Physical activity to prevent stroke mortality in Brazil (1990-2019)

**DOI:** 10.1590/0037-8682-0252-2021

**Published:** 2022-01-28

**Authors:** Diego Augusto Santos Silva, Antonio Luiz Pinho Ribeiro, Fatima Marinho, Mohsen Naghavi, Deborah Carvalho Malta

**Affiliations:** 1 Universidade Federal de Santa Catarina, Centro de Desportos, Departamento de Educação Física, Florianópolis, SC, Brasil.; 2 Universidade Federal de Minas Gerais, Faculdade de Medicina, Hospital das Clínicas, Programa de Pós-Graduação em Saúde Pública, Belo Horizonte, MG, Brasil.; 3 Vital Strategy, São Paulo, SP, Brasil.; 4 Institute for Health Metrics and Evaluation, Seattle, WA, USA.

**Keywords:** Epidemiology, Health survey, Physical activity, Sedentary behavior, Morbidity

## Abstract

**INTRODUCTION:**

This study aimed to estimate the burden of stroke mortality due to low levels of physical activity (PA) in Brazil from 1990 to 2019.

**METHODS:**

Data from the 2019 Global Burden of Disease (GBD) study for Brazil and Brazilian states were used. We used the number of deaths, age-standardized mortality rates, summary exposure value, and fraction of population risk attributable to low levels of PA. To standardize all estimates, data from the population aged 25 years or older were considered.

**RESULTS:**

The risk of exposure to low PA, SEV values, for the Brazilian male population was 11.8% (95%UI: 6.7; 19.9) and for the Brazilian female population was 13.2% (95%UI: 8.6; 19.2) in 2019. For males, it was estimated that there were, respectively, 2,025 (95%UI: 271; 4,839) and 3,595 (95%UI: 658; 7,302) deaths in 1990 and 2019 due to stroke attributable to low PA. For females, there were 2,518 (95%UI: 498; 5,006) and 4,735 (95%UI: 1,286; 8,495) deaths in 1990 and 2019 due to stroke attributable to low PA, respectively. From 1990 to 2019, reductions of 44.0% for males (95%UI: −0.54; -0.05) and 52.0% for females (95%UI: −0.60; -0.30) in age-standardized mortality rates due to stroke attributed to low PA were observed. Approximately 6.1% (for males) and 7.3% (for females) of deaths in 2019 due to stroke could be avoided if the Brazilian population were physically active.

**CONCLUSIONS:**

These findings support the promotion of PA in all Brazilian states for preventing early mortality due to stroke.

## INTRODUCTION

Stroke occurs when the blood supply to some region of the brain is interrupted or reduced, preventing brain tissues from receiving oxygen; therefore, leading to quick brain cell death^1^. This condition is considered a neurological disorder with heavy burden of morbidity and mortality around the world. In 2019, 6,552,724 deaths due to stroke were estimated in all age groups worldwide, and in Latin American and Caribbean countries, this estimate was 320,500 deaths^2^. In Brazil it was estimated that in 2013 there was 2,231.000 stroke and 568,000 stroke cases with severe disabilities. The point prevalence was 1.6% and 1.4% in men and women, respectively^3^. 

Due to the heavy burden of stroke mortality around the world, actions to prevent this neurological disorder are fundamental to the quality of life of the population. Among actions for stroke prevention, physical activity stands out in a preventive and therapeutic way in stroke survivors^4^. A systematic review with meta-analysis found 26 articles that analyzed the dose-response relationship between physical activity and stroke^5^. The authors found that for both sexes, meeting weekly physical activity guidelines led to a 16% lower risk of stroke than individuals with less weekly amount of physical activity. In addition, higher levels of physical activity further reduce the risk of stroke^5^. 

The biological mechanisms by which regular physical activity reduces the risk of stroke are several^6^. Physical activity reduces blood pressure, improves the lipid profile and decreases systemic inflammation, which result in decreased damage and atherosclerosis in brain blood vessels. In addition, physical activity improves the vasodilation and vasoconstriction properties of blood vessels and has antithrombotic effect, which reduces the risk of cerebrovascular events^5^. In this sense, the promotion of physical activity is justified and reduces the risk of stroke in all age groups.

The burden of stroke mortality attributable to low levels of physical activity has been little debated in Brazil. Many studies have focused on the burden of stroke mortality in the Brazilian population, but without focusing on a specific risk factor^7^ ^,^ ^8^. Studying the impact of a specific risk factor on the burden of mortality due to non-communicable diseases (NCD) helps evaluating and planning public health policies, and helps understanding how the population can benefit from actions that prevent these risk factors^9^. Thus, the aim of this study was to estimate the burden of stroke mortality due to low levels of physical activity in Brazil and Brazilian states from 1990 to 2019. 

## METHODS

### Study background

An analytical study based on estimates of the global burden of diseases for Brazil made by GBD 2019 was carried out, coordinated by the Institute for Health Metrics and Evaluation (IHME) in partnership with the Ministry of Health of Brazil^2^ ^,^ ^9^ ^-^ ^11^. In the mortality analysis, information from the Mortality Information System of the Ministry of Health was used, with adjustment for the underreporting of deaths and declaration of undefined/nonspecific causes, called garbage codes^2^ ^,^ ^9^.

### Stroke estimates

Stroke is defined as the rapid development of clinical signs of (usually focal) cerebral function disturbance lasting more than 24 hours or leading to death^12^. There are three stroke subtypes that can cause death (ischaemic; intracerebral haemorrhage; subarachnoid haemorrhage) being investigated in the GBD project^2^. The GBD project classifies causes into four levels, from the broadest (Level 1: non-communicable diseases), to the most specific (Level 4: intracerebral haemorrhage). Stroke is a Level-3 cause, while its subtypes are Level-4 causes.

The International Statistical Classification of Diseases (ICD), 10th revision (ICD-10) codes related to stroke have been mapped. ICD-10 codes for incidence, morbidity and mortality due to stroke were G45-G46.8, I60-I63.9, I65-I66.9, I67.0-I67.3, I67.5-I67.6, I68.1-I68.2, I69.0-I69.3^2^. In the present study, only ischaemic stroke estimates were considered (G45-G46.8, I63-I63.9, I65-I66.9, I67.2-I67.3, I67.5-I67.6, I69.3), because the evidence with physical activity is more consistent^5^. Additional information about these codes has been previously published^2^.

Using the Cause of Death Ensemble modeling (CODEm) approach with cause-specific covariates, mortality estimates for each individual cause were computed. CODEm is a flexible modelling tool that uses geospatial relationships and information 126 from covariates to produce death estimates for all locations across the time series (1990-2019)^2^. More details about these estimates are in literaure^2^ ^,^ ^9^. 

### Physical activity estimates

 General adult population surveys using random sampling procedures and evaluating self-reported physical activity in all life domains (leisure/recreation, work, household and commuting) were included. For global estimates, data were primarily derived from two standardized questionnaires, the Global Physical Activity Questionnaire (GPAQ) and the International Physical Activity Questionnaire (IPAQ), although all other surveys that evaluated PA intensity, frequency and duration performed across all activity domains were included^8^. In the case of Brazil, surveys such the Telephone-based Surveillance of Risk and Protective Factors for Chronic Diseases, Brazil World Health Survey, the National Health Survey, and other surveys are the basis for estimating national prevalence of physical activity^9^.

Physical activity frequency, duration and intensity were used to calculate the total metabolic equivalent (MET) - minutes per week. Firstly, level of physical activity was categorized by total MET-minutes per week using four categories based on rounded values closest to global distribution quartiles of total MET-minutes/week. The lowest limit for the Level 1 category (600 MET-min/week) is the recommended minimum amount of physical activity to obtain any health benefit^13^. We used four categories with higher thresholds rather than the GPAQ and IPAQ recommended 3 categories to better capture any additional protective effects from higher activity levels: Level 0: < 600 MET-min/week (inactive); Level 1: 600-3999 MET-min/week (low-active); Level 2: 4000-7,999 MET-min/week (moderately-active); Level 3: ≥ 8,000 MET-min/week (highly active). The theoretical minimum-risk exposure level (TMREL) for physical inactivity is 3000-4500 MET-min per week, which was calculated as the exposure at which minimal deaths are observed across outcomes^5^. A dose-response meta-analysis of prospective cohort studies was used to estimate the effect size of the change in levels of physical activity on ischemic stroke^5^. More details about these estimates are found in literaure^9^. 

The proportion of each year/age/sex subpopulation in each of the above four levels of PA was then estimated using 12 separate Dismod models (DisMod-MR 2.1 software, World Health Organization©, Geneva, Switzerland). DisMod-MR is a Bayesian geospatial disease modelling software that uses data on various disease parameters, epidemiological relationships among these parameters and geospatial relationships to produce prevalence and incidence estimates^2^ ^,^ ^9^. Using microdata on total MET-mins per week from individual-level surveys, the distribution of the level of physical activity at population level was characterized. We then used an ensemble approach to distribution fitting, borrowing characteristics from individual distributions to tailor a unique distribution to fit the data using a weighting scheme. The standard deviation of the level of physical activity of each population was characterized through linear regression that captured the relationship between standard deviation and mean levels of physical activity in nationally representative surveys^9^. 

To standardize all estimates of low levels of physical activity in Brazil, data from the population aged 25 years or older were considered. 

### Metrics and statistical analysis

Incident ischemic stroke cases (Additional file 1), summary exposure value (SEV) to low levels of physical activity (Additional file 2), absolute number of deaths, mortality rates (per 100,000 inhabitants-crude and age-standardized), and population-attributable fraction (PAF)^9^ of deaths due to stroke related to low levels of physical activity were used as metrics.

 The counterfactual level of risk exposure used is the risk exposure that is both theoretically possible and minimizes risk in the exposed population that consequently captures the maximum population attributable burden^9^. For each risk evaluated in GBD study, included low physical activity, has been used the best available epidemiological evidence from published and unpublished relative risks by level of exposure and the lowest observed level of exposure from cohorts used to select a single level of risk exposure combined to establish the TMREL. For this reason, the population attributable fraction (PAF) was estimated, which represents the proportion of risk that would be reduced in a given year if the exposure to a risk factor in the past was reduced to an ideal exposure scenario^9^. To calculate PAF for each risk factor, the GBD study relies on evidence extracted from literature, randomized controlled trials with sufficient sample size, cohort studies and other^9^.

SEV represents the measure of a population’s exposure to a risk factor that takes into account the extent of exposure by risk level and the severity of its contribution to the burden of the disease^9^. SEV takes value zero when there is no excess risk for a given population and value one when the population is at the highest risk level. SEV is reported in this study on a scale from 0 to 100% to emphasize that it is a risk-weighted prevalence, which measure was standardized by age. 

To standardize all estimates of low levels of physical activity and ischemic stroke in Brazil, data from the population aged 25 years or older were considered. In the tables/figures of this article, for better visualization, information was presented for the years 1990, 2010 and 2019; however, for the calculation of changes over time by year, the entire historical series from 1990 to 2019 was considered. In the tables of the article we showed estimates of the deaths due to stroke attributed to low levels of physical activity with 95% uncertainty intervals (UIs). More details on the term UIs can be found in the literature^14^. More details of historical series are available in literature^2^ ^,^ ^9^.

For this article, the following statistical software were used: DisMod-MR 2.1 Software (World Health Organization©, Geneva, Switzerland), Stata Statistical Software 15.0 (StataCorp©, Texas, United States of America), Microsoft Excel 12.0 (Microsoft©, Redmond, United States of America). 

## RESULTS

In the Brazilian male population, 100,950 (95% UI: 89,558; 114,786) incident stroke cases were estimated in 1990, 119,009 (95% UI: 106,596; 134,814) in 2010, and 138,785 (95% UI: 122,901; 159,256) in 2019. In the Brazilian female population, 97,135 (95% UI: 86,978; 108,860) incident stroke cases were estimated in 1990, 120,044 (95% UI: 108,600; 133,568) in 2010, and 141,053 (95% UI: 126,485; 158,450) in 2019. Information of incident cases and age-standardized incident stroke cases by Brazilian states are in Additional file 1 ( Supplementary Table 1, 2 and 3). The risk of exposure to low levels of physical activity, SEV, for the Brazilian male population was 11.4% (95% UI: 6.3; 19.5) in 1990, 12.0% (95% UI: 7.1; 19.8) in 2010, and 11.8% (95% UI: 6.7; 19.9) in 2019. This risk of exposure was similar in 1990, 2010 and 2019. The risk of exposure to low levels of physical activity for the Brazilian female population was 12.6% (95% UI: 8.0; 19.1) in 1990, 13.4% (95% UI 8.7; 19.3) in 2010, and 13.2% (95% UI: 8.6; 19.2) in 2019. This risk of exposure was similar in 1990, 2010 and 2019. Information of SEV values by Brazilian states is in Additional file 1 ( Supplementary Table 4). 

In the Brazilian male population, 2,025 (95% UI: 271; 4,839), 3,061 (95% UI: 559; 6,306) and 3,595 (95% UI: 658; 7,302) deaths due to stroke attributable to low levels of physical activity were estimated in 1990, 2010 and 2019, respectively. In the Brazilian female population, 2,518 (95% UI: 498; 5,006), 3,988 (95% UI: 1,123; 7,107) and 4,735 (95% UI: 1,286; 8,495) deaths due to stroke attributable to low levels of physical activity were estimated in 1990, 2010 and 2019, respectively. Table 1 shows information on mortality due to stroke attributed to low levels of physical activity per Brazilian state. 

**TABLE 1:** Number of deaths due to stroke attributable to low physical activity in Brazil and Brazilian states in 1990, 2010, and 2019 in ages ≥ 25 years.

	Male						Female					
	1990		2010		2019		1990		2010		2019	
	n	(95% UI)	n	(95% UI)	n	(95% UI)	n	(95% UI)	n	(95% UI)	n	(95% UI)
Brazil	2,025	(271; 4,839)	3,061	(559; 6,306)	3,595	(658; 7,302)	2,518	(498; 5,006)	3,988	(1,123; 7,107)	4,735	(1,286; 8,495)
Acre	03	(00; 07)	08	(01; 15)	08	(01; 15)	03	(01; 06)	07	(02; 13)	10	(03; 19)
Alagoas	41	(05; 99)	55	(10; 119)	55	(10; 119)	50	(10; 99)	76	(21; 137)	96	(26; 175)
Amapá	02	(00; 04)	04	(01; 09)	04	(01; 09)	02	(00; 04)	05	(01; 09)	08	(02; 15)
Amazonas	14	(02; 33)	31	(06; 61)	31	(06; 61)	19	(04; 37)	36	(11; 62)	51	(16; 92)
Bahia	134	(17; 334)	246	(41; 534)	246	(41; 534)	205	(41; 402)	227	(65; 406)	375	(101; 686)
Ceará	100	(14; 219)	189	(43; 369)	189	(43; 369)	107	(23; 213)	25	(06; 48)	264	(71; 482)
Distrito Federal	08	(01; 21)	23	(04; 47)	23	(04; 47)	10	(02; 21)	70	(16; 130)	37	(09; 69)
Espírito Santo	42	(06; 101)	64	(13; 133)	64	(13; 133)	44	(09; 91)	77	(21; 140)	83	(19; 159)
Goiás	44	(05; 116)	59	(10; 131)	59	(10; 131)	51	(10; 107)	165	(44; 300)	110	(29; 205)
Maranhão	63	(08; 161)	150	(28; 302)	150	(28; 302)	52	(10; 107)	33	(09; 58)	194	(50; 356)
Mato Grosso	14	(02; 37)	30	(05; 68)	30	(05; 68)	13	(02; 26)	35	(09; 63)	46	(13; 82)
Mato Grosso do Sul	18	(02; 45)	32	(05; 69)	32	(05; 69)	17	(03; 35)	402	(104; 725)	46	(12; 86)
Minas Gerais	227	(29; 551)	310	(60; 641)	310	(60; 641)	264	(45; 537)	106	(29; 194)	459	(120; 835)
Pará	51	(07; 122)	101	(21; 202)	101	(21; 202)	68	(13; 135)	122	(38; 211)	141	(37; 255)
Paraíba	59	(08; 127)	99	(21; 189)	99	(21; 189)	72	(14; 141)	217	(54; 394)	125	(38; 223)
Paraná	138	(17; 344)	182	(30; 390)	182	(30; 390)	140	(27; 290)	201	(56; 371)	271	(68; 502)
Pernambuco	117	(15; 266)	150	(27; 314)	30	(05; 68)	151	(28; 301)	83	(25; 146)	229	(60; 417)
Piauí	36	(04; 88)	62	(10; 129)	62	(10; 129)	39	(08; 78)	425	(127; 756)	106	(29; 187)
Rio de Janeiro	255	(34; 594)	305	(55; 613)	305	(55; 613)	355	(70; 691)	56	(15; 102)	459	(129; 825)
Rio Grande do Norte	32	(04; 75)	39	(06; 86)	39	(06; 86)	36	(07; 72)	338	(95; 606)	67	(19; 120)
Rio Grande do Sul	138	(17; 329)	201	(35; 421)	201	(35; 421)	219	(42; 431)	15	(04; 27)	397	(103; 718)
Rondônia	07	(01; 19)	20	(04; 39)	20	(04; 39)	05	(01; 10)	02	(01; 04)	24	(06; 45)
Roraima	01	(00; 03)	02	(00; 05)	02	(00; 05)	01	(00; 02)	227	(65; 406)	05	(01; 09)
Santa Catarina	66	(09; 154)	90	(16; 180)	90	(16; 180)	82	(16; 165)	120	(33; 212)	153	(43; 280)
São Paulo	385	(51; 969)	556	(96; 1,191)	556	(96; 1,191)	482	(97; 977)	778	(225; 1,403)	903	(238; 1,671)
Sergipe	20	(03; 48)	31	(05; 65)	31	(05; 65)	25	(05; 50)	38	(09; 72)	49	(12; 91)
Tocantins	08	(01; 19)	22	(04; 45)	22	(04; 45)	07	(01; 15)	18	(04; 33)	27	(07; 52)

**UI:** uncertainty interval.

Age-standardized mortality rates (per 100,000 inhabitants) due to stroke attributed to low levels of physical activity in the Brazilian male population were 7.5 (95% UI: 1.0; 16.9) in 1990, 5.0 (95% UI: 0.9; 10.1) in 2010 and 4.2 (95% UI: 0.8; 8.3) in 2019. In the Brazilian female population, age-standardized mortality rates (per 100,000 inhabitants) due to stroke attributed to low levels of physical activity were 7.7 (95% UI: 1.6; 14.9) in 1990, 4.5 (95% UI: 8.0; 1.3) in 2010 and 3.7 (95% UI: 6.6; 0.8) in 2019. Table 2 shows information on age-standardized mortality rates due to stroke attributed to low levels of physical activity per Brazilian state.

**TABLE 2:** Age-standardized mortality rate (per 100,000 inhabitants) due to stroke attributable to low physical activity in Brazil and Brazilian states in 1990, 2010, and 2019 in ages ≥ 25 years.

	Male						Female					
	1990		2010		2019		1990		2010		2019	
	Rate*	(95% UI)	Rate*	(95% UI)	Rate*	(95% UI)	Rate*	(95% UI)	Rate*	(95% UI)	Rate*	(95% UI)
Brazil	7.5	(1.0; 16.9)	5.0	(0.9; 10.1)	4.2	(0.8; 8.3)	7.7	(1.6; 14.9)	4.5	(1.3; 8.0)	3.7	(1.0; 6.6)
Acre	9.1	(1.4; 19.3)	5.6	(1.1; 10.7)	5.7	(1.1; 11.0)	6.8	(1.5; 12.9)	4.2	(1.1; 7.4	4.1	(1.1; 7.3)
Alagoas	8.9	(1.1; 20.6)	5.8	(1.0; 12.3)	5.4	(1.0; 11.8)	9.5	(2.0; 18.6)	6.1	(1.7; 10.9)	5.8	(1.6; 10.5)
Amapá	6.5	(0.9; 14.4)	4.4	(0.9; 9.0)	4.5	(0.9; 9.1)	6.1	(1.3; 11.7)	3.8	(1.1; 6.9)	3.8	(1.0; 7.0)
Amazonas	6.2	(0.8; 13.6)	4.5	(0.9; 8.7)	4.2	(0.9; 8.2)	8.5	(1.9; 15.9)	4.6	(1.5; 7.9)	4.1	(1.3; 7.3)
Bahia	5.4	(0.7; 13.1)	5.0	(0.8; 10.9)	4.5	(0.7; 9.4)	7.1	(1.4; 13.7)	4.2	(1.1; 7.6)	3.7	(1.0; 6.8)
Ceará	5.9	(0.9; 13.0)	5.8	(1.3; 11.2)	5.5	(1.1; 10.6)	5.5	(1.2; 10.9)	5.1	(1.4; 9.0)	4.6	(1.2; 8.5)
Distrito Federal	12.9	(1.9; 27.3)	7.8	(1.6; 14.9)	6.2	(1.5; 12.1)	9.3	(1.8; 18.2)	5.8	(1.5; 10.6)	4.8	(1.2; 8.8)
Espírito Santo	9.6	(1.2; 21.7)	5.8	(1.2; 11.6)	4.7	(0.8; 9.6)	9.7	(1.9; 19.2)	4.9	(1.2; 9.1)	3.7	(0.8; 7.1)
Goiás	7.6	(0.9; 18.2)	3.7	(0.6; 7.8)	3.1	(0.5; 6.7)	8.8	(1.8; 17.6)	4.7	(1.3; 8.3)	3.6	(1.0; 6.7)
Maranhão	12.0	(1.5; 27.5)	6.7	(1.3; 13.4)	8.7	(1.5; 17.3)	4.5	(0.9; 9.3)	5.2	(1.4; 9.4)	5.0	(1.3; 9.3)
Mato Grosso	6.1	(0.7; 14.5)	3.8	(0.6; 8.3)	2.9	(0.4; 6.4)	6.7	(1.4; 13.2)	4.6	(1.3; 8.0)	3.7	(1.0; 6.5)
Mato Grosso do Sul	6.4	(0.8; 15.3)	4.5	(0.8; 9.3)	3.5	(0.6; 7.2)	7.2	(1.4; 14.3)	4.4	(1.2; 7.9)	3.4	(0.9; 6.3)
Minas Gerais	7.8	(1.1; 17.6)	4.3	(0.8; 8.6)	3.3	(0.6; 6.6)	7.8	(1.5; 15.4)	4.0	(1.0; 7.2)	3.0	(0.8; 5.5)
Pará	8.5	(1.2; 19.2)	5.6	(1.2; 11.1)	5.2	(1.0; 10.5)	9.7	(1.9; 18.9)	5.0	(1.4; 9.1)	4.3	(1.1; 7.7)
Paraíba	5.9	(0.9; 12.6)	5.8	(1.2; 11.1)	4.4	(0.9; 8.4)	6.9	(1.4; 13.4)	4.9	(1.5; 8.5)	4.1	(1.2; 7.3)
Paraná	9.7	(1.3; 22.4)	5.5	(0.9; 11.3)	4.5	(0.7; 9.6)	10.2	(2.0; 20.1)	5.3	(1.4; 9.6)	4.2	(1.1; 7.8)
Pernambuco	7.9	(1.1; 17.3)	5.0	(0.9; 10.3)	4.9	(0.9; 10.0)	8.4	(1.7; 16.3)	4.7	(1.3; 8.6)	4.3	(1.1; 7.7)
Piauí	9.2	(1.2; 21.3)	5.4	(0.9; 11.1)	4.6	(0.8; 9.4)	8.0	(1.6; 15.5)	5.1	(1.5; 8.9)	4.6	(1.3; 8.2)
Rio de Janeiro	9.7	(1.4; 21.2)	5.6	(1.1; 11.0)	4.2	(0.9; 8.1)	9.4	(2.0; 17.8)	4.6	(1.4; 8.1)	3.5	(1.0; 6.2)
Rio Grande do Norte	4.9	(0.6; 11.4)	3.0	(0.5; 6.7)	2.8	(0.4; 6.2)	4.9	(1.0; 9.6)	3.1	(0.8; 5.6)	2.7	(0.7; 4.9)
Rio Grande do Sul	7.8	(0.9; 17.4)	5.1	(1.0; 10.4)	4.2	(0.7; 8.5)	8.7	(1.7; 16.7)	5.3	(1.5; 9.5)	4.2	(1.1; 7.7)
Rondônia	11.9	(1.8; 25.6)	5.2	(1.2; 9.8)	4.9	(1.2; 9.4)	14.3	(3.0; 26.9)	5.1	(1.4; 9.0)	4.2	(1.1; 7.7)
Roraima	8.9	(1.2; 20.2)	4.7	(0.8; 9.3)	4.6	(0.8; 8.9)	9.3	(2.0; 17.4)	5.4	(1.5; 9.2)	4.8	(1.4; 8.2)
Santa Catarina	9.2	(1.3; 20.4)	5.5	(1.0; 10.5)	4.2	(0.9; 8.1)	10.3	(2.1; 20.1)	5.1	(1.5; 9.0)	4.0	(1.1; 7.3)
São Paulo	7.0	(0.9; 16.6)	4.5	(0.8; 9.3)	3.4	(0.6; 7.0)	7.3	(1.5; 14.3)	4.1	(1.2; 7.3)	3.1	(0.8; 5.7)
Sergipe	8.9	(1.1; 19.9)	5.0	(0.9; 10.5)	4.7	(0.9; 9.7)	8.8	(1.7; 17.5)	4.3	(1.0; 8.0)	4.0	(1.0; 7.5)
Tocantins	9.3	(1.2; 21.7)	4.9	(0.9; 9.9)	6.1	(1.1; 12.2)	8.8	(1.9; 17.0)	4.1	(1.0; 7.4)	3.9	(1.0; 7.6)

**UI:** uncertainty interval; * age-standardized.

For males, there was stability in age-standardized mortality rates due to stroke attributed to low levels of physical activity from 1990 to 2010 (- 0.34%; 95% UI: - 0.32; 0.79). However, in Brazilian states of Roraima, Rondônia, Tocantins, Sergipe, Goiás and Paraná, decrease in age-standardized mortality rates due to stroke attributed to low levels of physical activity was observed. For the others states, there was stability in age-standardized mortality rates due to stroke attributed to low levels of physical activity. From 2010 to 2019, decrease in age-standardized mortality rates due to stroke attributed to low levels of physical activity was observed in Brazil (− 0.16%; 95% UI − 0.22; - 0.09), and in Brazilian states of Mato Grosso, Mato Grosso do Sul, Paraíba, Minas Gerais, São Paulo, Rio de Janeiro and Santa Catarina, and stability for the others states. From 1990 to 2019, decrease in age-standardized mortality rates due to stroke attributed to low levels of physical activity was observed in Brazil (− 0.44%; 95% UI − 0.54; - 0.05), and in Brazilian states of Roraima, Rondônia, Tocantins, Piauí, Rio Grande do Norte, Sergipe, in all states of Mid-Western, Southeastern, and Southern regions of Brazil, and stability for the other states (Figure 1). 

**FIGURE:**
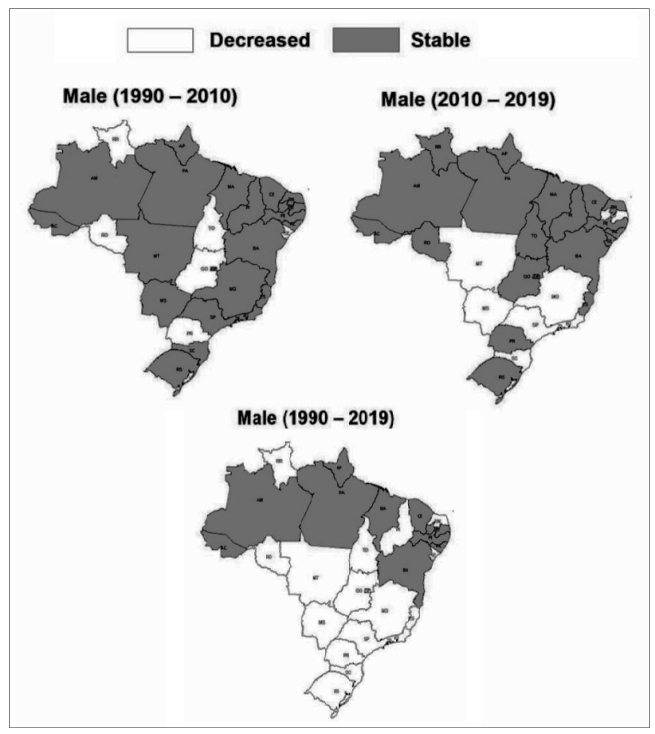
1: Change in age-standardized mortality rate (per 100,000 inhabitants) due to stroke attributable to low physical activity in men (≥ 25 years old) from Brazil (1990-2010; 2010-2019; and 1990-2019).

For females, decrease in age-standardized mortality rates due to stroke attributed to low levels of physical activity was observed from 1990 to 2010 in Brazil (− 0.41%; 95% UI − 0.50; - 0.12), in Brazilian states of Goiás, Mato Grosso do Sul, Pernambuco, Sergipe, Bahia, in the Federal District, and in all Brazilian states of the Northern, Southeastern, and Southern regions. Stability was observed for the other states. From 2010 to 2019, decrease in age-standardized mortality rates due to stroke attributed to low levels of physical activity was observed in Brazil (− 0.18%; 95% UI − 0.24; - 0.13), and in Brazilian states of Paraíba, Santa Catarina, Rio Grande do Sul, and in all Brazilian states of the Mid-Western and Southeastern regions. Stability was observed for the other states. From 1990 to 2019, decrease in age-standardized mortality rates due to stroke attributed to low levels of physical activity was observed in Brazil (− 0.52%; 95% UI − 0.60; - 0.30), and in all Brazilian states, except for states of Alagoas, Ceará and Maranhão, which showed stability in age-standardized mortality rates due to stroke attributed to low levels of physical activity (Figure 2). 

**FIGURE:**
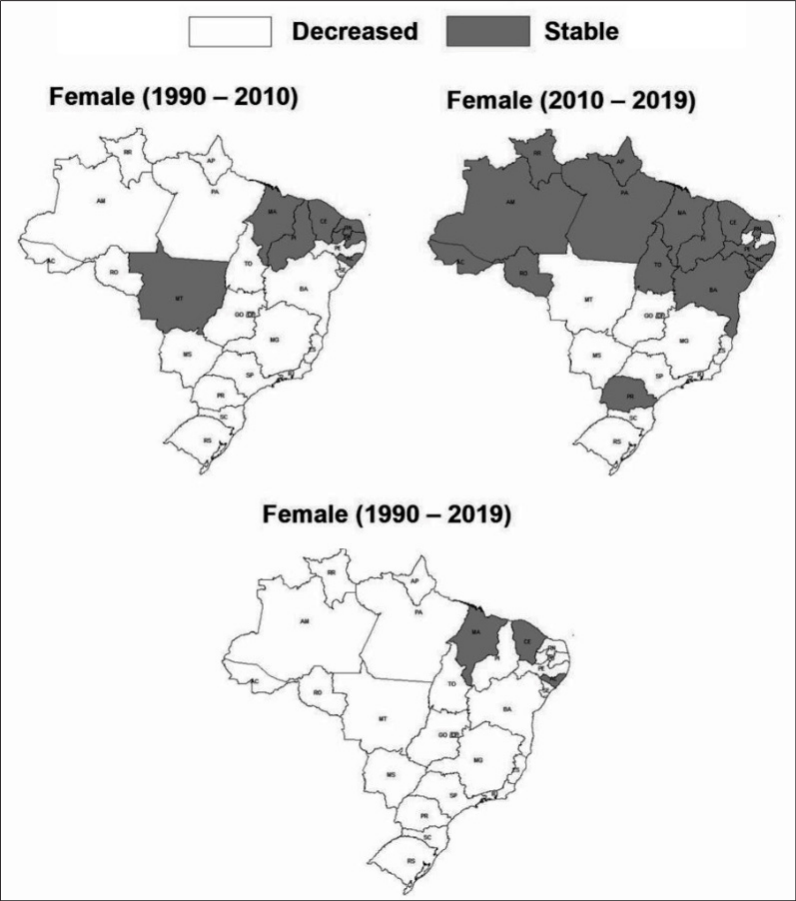
2: Change in age-standardized mortality rate (per 100,000 inhabitants) due to stroke attributable to low physical activity in women (≥ 25 years old) from Brazil (1990-2010; 2010-2019; and 1990-2019).

Age-standardized mortality rates (per 100,000 inhabitants) due to stroke attributable to low levels of physical activity was higher at more advanced ages compared to younger individuals. Approximately 4.8% in 1990, 6.0% in 2010, and 6.1% in 2019 of deaths due to stroke could be avoided if the Brazilian male population were physically active (e.g., PAF). In females, 6.2% in 1990, 7.3% in 2010, and 7.3% in 2019 of deaths due to stroke could be avoided if the Brazilian population were physically active (e.g., PAF) (Table 3). In addition, the information on the states by age and sex are in the Supplementary Tables (i.e., Supplementary Table 5; Supplementary Table 6; Supplementary Table 7; Supplementary Table 8; Supplementary Table 9; Supplementary Table 10). 

**TABLE 3:** Mortality rate (per 100,000 inhabitants) due to stroke attributable to low physical activity, and population attributable fraction in Brazil according to age in 1990, 2010, and 2019.

	1990		2010		2019	
	Rate* (95% UI)	PAF (95% UI)	Rate* (95% UI)	PAF (95% UI)	Rate* (95% UI)	PAF (95% UI)
	Male					
**25-49 years**	0.2 (0.1; 0.8)	1.2 (0.1; 4.2)	0.1 (0.0; 0.3)	1.0 (0.1; 3.6)	0.1 (0.0; 0.3)	1,0 (0.0; 3.4)
**50-69 years**	6.5 (0.5; 19.2)	2.2 (0.2; 6.7)	3.0 (0.3; 8.7)	2.2 (0.2; 6.1)	2.6 (0.2; 7.1)	2,2 (0.2; 6.2)
**70+ years**	76.9 (10.2; 168.3)	5.7 (0.8; 12.5)	65.1 (13.1; 127.3)	7.6 (1.5; 14.6)	55.3 (11.0; 106.7)	7,8 (1.5; 14.8)
	Female					
**25-49 years**	0.2 (0.1; 0.5)	1.2 (0.1; 3.3)	0.1 (0.1; 0.2)	0.9 (0.1; 2.5)	0.1 (0.0; 0.2)	0,9 (0.1; 2.5)
**50-69 years**	4.0 (0.4; 9.8)	2.1 (0.2; 5.2)	1.6 (0.2; 3.8)	1.8 (0.2; 4.3)	1.4 (0.2; 3.1)	1,9 (0.2; 4.4)
**70+ years**	90.3 (18.9; 173.1)	7.6 (1.6; 14.5)	68.3 (19.9; 118.6)	9.8 (2.9; 17.0)	58.3 (16.4; 102.9)	9,8 (2.8; 16.8)

PAF: population attributable fraction; UI: uncertainty interval; *Rate per 100,000 inhabitant.

## DISCUSSION

The present study has the originality of presenting information on stroke mortality due to low levels of physical activity in Brazil. This information was systematized to bring evidence from all Brazilian states and highlight the importance of physical activity in the prevention of stroke.

The main finding of this research was that from 1990 to 2019, decrease in age-standardized mortality rates due to stroke attributable to low levels of physical activity was observed in the Brazilian population aged ≥25 years. However, this decrease was not observed when Brazilian states were analyzed separately because some states of the Northern and Northeastern regions showed stability in age-standardized mortality rates due to stroke attributable to low levels of physical activity from 1990 to 2019. In addition, it was observed that approximately 6.0% and 7.0% of deaths due to stroke could be avoided with regular physical activity in men and women, respectively.

As in other studies^15^ ^,^ ^16^, this study highlighted the health consequences of social and economic inequalities among the different regions of Brazil. The Northern and Northeastern regions of Brazil have historically suffered from income inequality, illiteracy, urban violence, and reduced access to public health services^15^. All these conditions make it difficult for the population to have full and universal access to adequate treatment in cases of stroke, which justify the lack of reduction in age-standardized mortality rates due to stroke attributable to low levels of physical activity over the years. 

The reduction in stroke mortality is linked to the incidence and lethality of the disease^7^. Incidence is related to stroke risk factors, while lethality assesses the effectiveness of treatment applied to the population. The control of risk factors and the improvement of the population's socioeconomic conditions can reduce mortality rates^7^. From 1990 to 2019, improvements in the socioeconomic conditions of the Brazilian population were observed, which resulted in equitable decrease in age-standardized mortality rates due to stroke attributable to low levels of physical activity by Brazilian region. However, as demonstrated by Silva^17^ and Teixeira and Paim^16^, the Northern and Northeastern regions of Brazil have not improved in the same magnitude in terms of socioeconomic conditions and in relation to access to health services with high technology compared to other Brazilian regions. This study showed that the risk of the Brazilian population in relation to low levels of physical activity has been stable over the years (SEV) (but the ideal scenario is further improvements in the levels of physical activity for the population). The stability observed in SEV can be due to public policies implemented in Brazil especially after 2006 to improve the levels of physical activity in the population^11^ ^,^ ^18^ ^-^ ^20^. 

The stability in the risk of exposure of the Brazilian population to low levels of physical activity and improvements in the living conditions from 1990 to 2019 can be a justification for the decrease in age-standardized mortality rates due to stroke attributable to low levels of physical activity in most Brazilian states. However, the effectiveness of the stroke treatment applied to the population is not similar among Brazilian states. Garritano et al.^7^ pointed out that for the control of morbidity and mortality caused by stroke, it is necessary to apply high-tech procedures, such as angioplasty, a greater amount of equipment in hospitals for more accurate diagnosis, such as computed tomography scan, nuclear magnetic resonance, and faster attendance by health services. 

This study also demonstrated that as the age group of the population increased the age-standardized mortality rates due to stroke attributable to low levels of physical activity also increased and more physical activity could have prevented stroke mortality. The benefits of regular physical activity are numerous for all age groups^6^. In older adults, in particular, the practice of regular physical activity attenuates the effects of age on the decline of physiological functions, which can decrease, for example, cases of stroke in this age group^6^.

This study has many limitations that need to be highlighted. The first one is that physical activity was estimated from surveys that used self-reported measures and not objective measures. Objective measures are more accurate in estimating the daily amount of physical activity than self-reported measures; however, in epidemiological studies, self-reported measures are the most widely used^10^. The second one was that this research considered the practice of physical activity in the four domains and did not specify the contribution of each physical activity domain in relation to stroke. It is relevant to check the contribution of each physical activity domain in the prevention of NCDs for better targeting of public health actions^19^. The third one was that this study only determined the burden of mortality due to ischemic stroke, not investigating other types of stroke, such as the intracerebral hemorrhagic, which is defined as stroke with a focal collection of blood in the brain not due to trauma, and the subarachnoid hemorrhagic, which is defined as non-traumatic stroke due to bleeding into the subarachnoid space of the brain^12^.

It could be concluded that low levels of physical activity contributed to a substantial number of deaths by ischemic stroke in Brazil and in the different Brazilian states from 1990 to 2019. From 1990 to 2019, decrease in age-standardized mortality rates due to ischemic stroke attributable to low levels of physical activity was observed in Brazil. Brazilian states with the highest social inequalities showed lower reductions (from 1990 to 2019) in age-standardized mortality rates due to stroke attributable to low levels of physical activity.
