# Supplementary material for: Physical activity to prevent stroke mortality in Brazil (1990-2019)
Source: Rev Soc Bras Med Trop. 2022 Jan 28;55(Suppl 1):e0252-2021. doi: 10.1590/0037-8682-0252-2021 (PMC9020380; doi:10.1590/0037-8682-0252-2021)
Supplement: Supplementary file 1 [file 1678-9849-rsbmt-55-s01-e0252-2021-supp1.pdf]

SUPPLEMENTARY TABLE 1: Incidence of stroke in the Brazilian population aged ≥ 25 years in 1990, 2010, and 2019.

	Male									Female								
	1990			2010			2019			1990			2010			2019		
	n	(95% U.I.)		n	(95% U.I.)		n	(95% U.I.)		n	(95% U.I.)		n	(95% U.I.)		n	(95% U.I.)	
Brazil	100,950	89,558	114,786	119,009	106,596	134,814	138,785	122,901	159,256	97,135	86,978	108,860	120,044	108,600	133,568	141,053	126,485	158,450
Acre	189	166	216	317	280	361	413	363	474	163	145	182	295	264	330	390	349	441
Alagoas	1,599	1,403	1,841	2,015	1,782	2,312	2,316	2,030	2,663	1,560	1,393	1,761	2,058	1,854	2,301	2,412	2,140	2,713
Amapá	107	94	121	246	218	278	357	313	414	100	89	113	217	194	242	331	294	373
Amazonas	893	786	1,014	1,412	1,257	1,610	1,886	1,664	2,159	793	706	892	1,275	1,144	1,420	1,713	1,525	1,918
Bahia	7,211	6,361	8,282	8,824	7,820	10,071	10,008	8,749	11,502	7,405	6,595	8,345	9,410	8,463	10,486	10,858	9,693	12,243
Ceará	3,627	3,207	4,161	5,173	4,598	5,911	6,020	5,302	6,926	3,749	3,348	4,197	5,432	4,877	6,102	6,262	5,568	7,080
Distrito Federal	723	633	825	1,104	979	1,254	1,424	1,252	1,630	761	679	851	1,178	1,058	1,322	1,522	1,349	1,708
Espírito Santo	1,904	1,671	2,191	2,401	2,130	2,725	2,744	2,418	3,173	1,754	1,565	1,979	2,312	2,073	2,586	2,708	2,409	3,084
Goiás	2,650	2,342	3,026	3,327	2,953	3,783	4,172	3,647	4,800	2,290	2,039	2,559	3,075	2,763	3,458	3,919	3,489	4,419
Maranhão	2,694	2,368	3,090	3,937	3,469	4,479	4,333	3,804	4,979	2,358	2,105	2,650	3,841	3,446	4,289	4,433	3,949	4,986
Mato Grosso	1,026	902	1,175	1,735	1,544	1,971	2,156	1,899	2,479	789	702	885	1,400	1,256	1,562	1,821	1,630	2,047
Mato Grosso do Sul	1,160	1,026	1,319	1,540	1,369	1,756	1,869	1,635	2,158	956	851	1,070	1,380	1,235	1,546	1,745	1,556	1,966
Minas Gerais	12,212	10,859	13,887	13,829	12,289	15,665	15,764	13,795	18,160	11,362	10,137	12,728	13,390	11,976	15,013	15,411	13,701	17,388
Pará	2,424	2,147	2,763	3,769	3,351	4,280	4,705	4,141	5,386	2,255	2,011	2,529	3,395	3,051	3,771	4,290	3,820	4,849
Paraíba	2,093	1,836	2,408	2,417	2,153	2,717	2,722	2,403	3,109	2,147	1,913	2,420	2,586	2,334	2,881	2,879	2,588	3,230
Paraná	6,546	5,754	7,533	7,394	6,495	8,418	8,454	7,407	9,725	5,668	5,016	6,409	6,938	6,203	7,826	8,155	7,236	9,247
Pernambuco	4,715	4,130	5,402	5,372	4,762	6,141	6,184	5,425	7,052	5,081	4,512	5,729	5,841	5,220	6,535	6,689	5,970	7,597
Piauí	1,451	1,269	1,681	2,008	1,779	2,284	2,378	2,086	2,744	1,350	1,207	1,515	2,044	1,833	2,284	2,357	2,100	2,648
Rio de Janeiro	11,811	10,369	13,535	11,659	10,348	13,285	12,975	11,400	15,005	12,504	11,189	14,228	13,100	11,791	14,744	14,432	12,913	16,350
Rio Grande do Norte	1,503	1,317	1,729	1,746	1,557	1,969	2,131	1,875	2,432	1,481	1,320	1,668	1,809	1,623	2,022	2,200	1,958	2,474
Rio Grande do Sul	7,301	6,443	8,332	7,875	6,974	8,896	8,970	7,806	10,354	7,664	6,817	8,672	8,517	7,630	9,611	9,609	8,527	10,991
Rondônia	541	469	620	821	723	941	1,005	885	1,152	393	348	445	675	606	753	872	782	983
Roraima	85	75	97	165	146	188	249	218	288	57	50	64	132	118	148	197	174	222
São Paulo	22,283	19,485	25,361	24,267	21,645	27,539	28,775	25,324	33,333	20,549	18,178	23,093	24,168	21,648	27,016	29,158	25,971	32,817
Santa Catarina	2,877	2,534	3,299	3,706	3,342	4,148	4,428	3,866	5,100	2,675	2,384	3,017	3,644	3,305	4,032	4,314	3,869	4,832
Sergipe	849	745	981	1,154	1,015	1,316	1,413	1,237	1,623	878	781	990	1,243	1,114	1,379	1,525	1,360	1,721
Tocantins	477	419	549	798	710	917	934	818	1,067	392	347	438	689	625	764	853	763	958

U.I.: uncertainty interval
