# Supplementary material for: Physical activity to prevent stroke mortality in Brazil (1990-2019)
Source: Rev Soc Bras Med Trop. 2022 Jan 28;55(Suppl 1):e0252-2021. doi: 10.1590/0037-8682-0252-2021 (PMC9020380; doi:10.1590/0037-8682-0252-2021)
Supplement: Supplementary file 2 [file 1678-9849-rsbmt-55-s01-e0252-2021-supp2.pdf]

SUPPLEMENTARY TABLE 2: Incidence rate (per 100,000 inhabitants) and percentage of change in the incidence rate of stroke in the male Brazilian population in 1990, 2010, and 2019.

	1990			2010			2019			Change (1990-2010)			Change (2010-2019)			Change (1990-2019)		
	Rate*	95% U.I.		Rate*	95% U.I.		Rate*	95% U.I.		%*	95% U.I.		%*	95% U.I.		%*	95% U.I.	
Brazil	240.8	214.4	274.0	154.8	138.4	174.2	138.3	123.1	157.4	-0.4	-0.3	-0.4	-0.1	-0.1	-0.1	-0.4	-0.4	-0.4
Acre	218.5	193.6	248.3	157.7	139.9	180.0	143.0	126.1	162.4	-0.3	-0.2	-0.3	-0.1	0.0	-0.1	-0.3	-0.3	-0.4
Alagoas	256.1	225.9	293.9	187.8	166.7	215.3	169.5	149.2	194.7	-0.3	-0.2	-0.3	-0.1	0.0	-0.1	-0.3	-0.3	-0.4
Amapá	211.7	186.3	242.0	153.0	136.3	173.6	142.9	126.0	166.2	-0.3	-0.2	-0.3	-0.1	0.0	-0.1	-0.3	-0.3	-0.4
Amazonas	218.2	193.0	248.6	149.0	132.6	171.1	137.2	121.4	157.3	-0.3	-0.3	-0.4	-0.1	0.0	-0.1	-0.4	-0.3	-0.4
Bahia	231.9	205.3	265.5	162.1	143.8	185.0	147.2	129.3	168.9	-0.3	-0.3	-0.3	-0.1	0.0	-0.1	-0.4	-0.3	-0.4
Ceará	196.6	174.5	225.6	155.9	138.5	177.8	142.1	125.6	162.2	-0.2	-0.2	-0.2	-0.1	0.0	-0.1	-0.3	-0.2	-0.3
Distrito Federal	233.2	206.5	265.0	150.1	132.4	172.0	129.1	113.8	148.7	-0.4	-0.3	-0.4	-0.1	-0.1	-0.2	-0.4	-0.4	-0.5
Espírito Santo	264.6	232.4	303.2	167.4	148.4	190.5	144.5	128.4	166.5	-0.4	-0.3	-0.4	-0.1	-0.1	-0.2	-0.5	-0.4	-0.5
Goiás	241.1	214.0	274.7	144.9	128.7	163.8	132.2	116.5	151.5	-0.4	-0.4	-0.4	-0.1	0.0	-0.1	-0.5	-0.4	-0.5
Maranhão	231.6	205.5	263.0	167.1	147.5	190.5	150.8	133.5	172.7	-0.3	-0.2	-0.3	-0.1	0.0	-0.2	-0.3	-0.3	-0.4
Mato Grosso	223.1	197.3	253.8	154.7	137.7	176.2	133.4	118.1	152.9	-0.3	-0.3	-0.3	-0.1	-0.1	-0.2	-0.4	-0.4	-0.4
Mato Grosso do Sul	241.6	214.6	274.6	158.2	141.0	180.0	140.1	123.5	159.9	-0.3	-0.3	-0.4	-0.1	-0.1	-0.2	-0.4	-0.4	-0.5
Minas Gerais	261.0	233.0	296.6	158.5	141.0	179.3	137.8	121.5	157.2	-0.4	-0.4	-0.4	-0.1	-0.1	-0.2	-0.5	-0.4	-0.5
Pará	228.7	203.1	260.8	160.8	142.9	181.8	143.7	126.7	164.1	-0.3	-0.3	-0.3	-0.1	-0.1	-0.2	-0.4	-0.3	-0.4
Paraíba	204.9	181.4	233.3	152.0	135.5	171.0	137.9	122.0	156.9	-0.3	-0.2	-0.3	-0.1	0.0	-0.1	-0.3	-0.3	-0.4
Paraná	268.2	237.6	305.6	166.2	146.4	189.5	146.8	129.8	167.5	-0.4	-0.3	-0.4	-0.1	-0.1	-0.2	-0.5	-0.4	-0.5
Pernambuco	233.6	206.6	265.7	161.3	143.7	184.1	149.6	131.3	170.1	-0.3	-0.3	-0.4	-0.1	0.0	-0.1	-0.4	-0.3	-0.4
Piauí	220.9	194.5	254.9	161.6	143.6	183.1	145.7	128.5	167.3	-0.3	-0.2	-0.3	-0.1	-0.1	-0.2	-0.3	-0.3	-0.4
Rio de Janeiro	275.1	244.1	312.2	164.6	146.8	187.0	143.4	127.0	164.0	-0.4	-0.4	-0.4	-0.1	-0.1	-0.2	-0.5	-0.5	-0.5
Rio Grande do Norte	207.7	184.1	237.9	138.8	123.5	157.3	130.2	114.9	148.5	-0.3	-0.3	-0.4	-0.1	0.0	-0.1	-0.4	-0.3	-0.4
Rio Grande do Sul	245.5	218.0	279.2	155.0	137.6	176.1	139.5	122.7	159.5	-0.4	-0.3	-0.4	-0.1	0.0	-0.1	-0.4	-0.4	-0.5
Rondônia	245.8	217.6	279.7	152.0	134.7	174.4	134.2	118.4	152.6	-0.4	-0.3	-0.4	-0.1	-0.1	-0.2	-0.5	-0.4	-0.5
Roraima	221.6	196.3	253.0	142.3	126.6	161.3	130.7	115.6	150.1	-0.4	-0.3	-0.4	-0.1	0.0	-0.1	-0.4	-0.4	-0.4
São Paulo	233.1	205.3	267.1	140.6	125.7	158.1	127.4	113.1	145.5	-0.4	-0.4	-0.4	-0.1	0.0	-0.1	-0.5	-0.4	-0.5
Santa Catarina	233.2	206.5	265.9	147.8	132.9	164.8	126.5	111.6	143.6	-0.4	-0.3	-0.4	-0.1	-0.1	-0.2	-0.5	-0.4	-0.5
Sergipe	236.3	208.1	272.3	162.3	143.0	184.4	148.4	130.2	170.6	-0.3	-0.3	-0.4	-0.1	0.0	-0.1	-0.4	-0.3	-0.4
Tocantins		191.6	247.6	153.1	135.9	175.8	137.2	120.8	156.0	-0.	-0.	-0.3	-0.	0.0	-0.	**-0.**		-0.

U.I.: uncertainty interval; * age-standardized.
