# Supplementary material for: Physical activity to prevent stroke mortality in Brazil (1990-2019)
Source: Rev Soc Bras Med Trop. 2022 Jan 28;55(Suppl 1):e0252-2021. doi: 10.1590/0037-8682-0252-2021 (PMC9020380; doi:10.1590/0037-8682-0252-2021)
Supplement: Supplementary file 3 [file 1678-9849-rsbmt-55-s01-e0252-2021-supp3.pdf]

SUPPLEMENTARY TABLE 3: Incidence rate (per 100,000 inhabitants) and percentage of change in the incidence rate of stroke in the female Brazilian population in 1990, 2010, and 2019.

	1990			2010			2019			Change (1990-2010)			Change (2010-2019)			Change (1990-2019)		
	Rate*	95% U.I.		Rate*	95% U.I.		Rate*	95% U.I.		%*	95% U.I.		%*	95% U.I.		%*	95% U.I.	
Brazil	209.8	188.8	233.5	131.6	118.8	145.4	117.9	106.3	130.6	-0.4	-0.4	-0.4	-0.1	-0.1	-0.1	-0.4	-0.5	-0.4
Acre	200.6	179.8	225.0	139.4	125.5	155.6	124.7	111.6	140.9	-0.3	-0.3	-0.3	-0.1	-0.2	-0.1	-0.4	-0.4	-0.3
Alagoas	228.6	205.1	256.6	160.4	144.6	179.3	143.9	128.9	161.2	-0.3	-0.3	-0.3	-0.1	-0.1	-0.1	-0.4	-0.4	-0.3
Amapá	188.7	169.0	213.3	127.8	114.4	143.0	121.8	108.4	136.7	-0.3	-0.4	-0.3	0.0	-0.1	0.0	-0.4	-0.4	-0.3
Amazonas	198.1	177.3	221.9	129.6	116.3	144.7	118.1	105.4	132.1	-0.3	-0.4	-0.3	-0.1	-0.1	0.0	-0.4	-0.4	-0.4
Bahia	215.3	192.5	241.9	143.9	129.7	160.5	128.8	115.8	143.6	-0.3	-0.4	-0.3	-0.1	-0.1	-0.1	-0.4	-0.4	-0.4
Ceará	181.0	162.2	201.7	135.8	122.2	151.5	121.9	108.6	136.5	-0.2	-0.3	-0.2	-0.1	-0.1	-0.1	-0.3	-0.4	-0.3
Distrito Federal	212.5	190.6	237.3	127.2	114.3	141.6	107.9	96.3	120.2	-0.4	-0.4	-0.4	-0.2	-0.2	-0.1	-0.5	-0.5	-0.5
Espírito Santo	231.5	206.8	258.2	141.1	126.5	157.5	123.1	109.9	138.2	-0.4	-0.4	-0.4	-0.1	-0.2	-0.1	-0.5	-0.5	-0.4
Goiás	215.8	193.4	241.4	127.1	114.6	142.0	114.2	102.1	127.7	-0.4	-0.4	-0.4	-0.1	-0.1	-0.1	-0.5	-0.5	-0.4
Maranhão	178.4	159.7	199.8	146.1	131.4	162.8	130.4	116.9	146.1	-0.2	-0.2	-0.1	-0.1	-0.2	-0.1	-0.3	-0.3	-0.2
Mato Grosso	200.9	179.7	225.7	133.9	120.5	149.7	116.6	104.9	130.2	-0.3	-0.4	-0.3	-0.1	-0.2	-0.1	-0.4	-0.4	-0.4
Mato Grosso do Sul	213.7	191.3	237.7	135.5	122.0	151.1	121.5	109.2	135.5	-0.4	-0.4	-0.3	-0.1	-0.1	-0.1	-0.4	-0.5	-0.4
Minas Gerais	222.3	199.2	247.9	133.5	119.7	148.2	117.5	104.9	131.3	-0.4	-0.4	-0.4	-0.1	-0.2	-0.1	-0.5	-0.5	-0.4
Pará	210.8	188.2	236.4	140.2	126.1	155.6	122.9	109.7	138.0	-0.3	-0.4	-0.3	-0.1	-0.2	-0.1	-0.4	-0.4	-0.4
Paraíba	188.9	169.1	211.8	129.9	117.9	143.9	117.5	106.2	130.4	-0.3	-0.3	-0.3	-0.1	-0.1	-0.1	-0.4	-0.4	-0.3
Paraná	230.7	206.0	259.4	139.2	124.8	156.2	123.4	110.3	138.2	-0.4	-0.4	-0.4	-0.1	-0.2	-0.1	-0.5	-0.5	-0.4
Pernambuco	216.0	192.2	241.8	138.7	124.6	154.3	126.4	113.1	142.2	-0.4	-0.4	-0.3	-0.1	-0.1	0.0	-0.4	-0.4	-0.4
Piauí	191.4	171.7	213.0	139.9	125.6	155.8	123.2	110.4	137.1	-0.3	-0.3	-0.2	-0.1	-0.2	-0.1	-0.4	-0.4	-0.3
Rio de Janeiro	237.8	213.9	267.3	140.7	127.1	156.6	123.5	111.5	138.0	-0.4	-0.4	-0.4	-0.1	-0.2	-0.1	-0.5	-0.5	-0.5
Rio Grande do Norte	185.9	166.5	206.8	117.5	105.8	130.3	109.8	98.5	122.3	-0.4	-0.4	-0.3	-0.1	-0.1	0.0	-0.4	-0.4	-0.4
Rio Grande do Sul	220.1	196.8	248.1	135.1	121.6	151.1	122.1	108.8	137.8	-0.4	-0.4	-0.4	-0.1	-0.1	0.0	-0.4	-0.5	-0.4
Rondônia	226.9	202.2	256.4	135.5	121.8	150.8	118.2	105.9	132.8	-0.4	-0.4	-0.4	-0.1	-0.2	-0.1	-0.5	-0.5	-0.5
Roraima	194.7	174.1	219.6	124.6	112.0	139.7	111.8	99.5	125.0	-0.4	-0.4	-0.3	-0.1	-0.1	-0.1	-0.4	-0.5	-0.4
São Paulo	193.2	172.3	215.7	114.8	103.0	127.7	105.9	95.1	117.5	-0.4	-0.4	-0.4	-0.1	-0.1	0.0	-0.5	-0.5	-0.4
Santa Catarina	202.8	182.0	227.5	125.9	114.5	138.1	108.9	97.6	121.1	-0.4	-0.4	-0.3	-0.1	-0.2	-0.1	-0.5	-0.5	-0.4
Sergipe	212.9	190.7	239.4	141.6	126.9	157.2	129.1	115.2	144.4	-0.3	-0.4	-0.3	-0.1	-0.1	0.0	-0.4	-0.4	-0.4
Tocantins	199.9	179.0	223.4	139.7	127.0	154.6	123.1	109.9	137.3	-0.3	-0.3	-0.3	-0.1	-0.2	-0.1	-0.4	-0.4	-0.4

U.I.: uncertainty interval; * age-standardized.
