# Supplementary material for: Physical activity to prevent stroke mortality in Brazil (1990-2019)
Source: Rev Soc Bras Med Trop. 2022 Jan 28;55(Suppl 1):e0252-2021. doi: 10.1590/0037-8682-0252-2021 (PMC9020380; doi:10.1590/0037-8682-0252-2021)
Supplement: Supplementary file 4 [file 1678-9849-rsbmt-55-s01-e0252-2021-supp4.pdf]

SUPPLEMENTARY TABLE 4: Summary exposure values (SEV) for low physical activity in the Brazilian population aged ≥ 25 years in 1990, 2010, and 2019.

	Male									Female								
	1990			2010			2019			1990			2010			2019		
	SEV*	95% U.I.		SEV*	95% U.I.		SEV*	95% U.I.		SEV*	95% U.I.		SEV*	95% U.I.		SEV*	95% U.I.	
Brazil	11.4	6.3	19.5	12.0	7.1	19.8	11.8	6.7	19.9	12.6	8.0	19.1	13.4	8.7	19.3	13.2	19.2	8.6
Acre	12.3	6.7	20.9	12.8	7.5	20.9	12.9	7.7	20.9	13.4	8.5	19.7	14.5	9.8	20.6	14.5	20.3	9.7
Alagoas	10.4	5.2	19.2	11.0	6.2	19.0	10.9	6.3	18.8	13.1	8.2	19.7	14.2	9.4	20.3	14.1	20.1	9.3
Amapá	11.3	6.0	19.7	11.7	6.6	20.0	11.7	6.6	19.9	13.1	8.2	19.5	14.0	9.3	20.0	14.0	20.1	9.2
Amazonas	12.1	6.6	20.2	12.4	7.2	20.5	12.2	7.0	20.1	14.0	9.1	20.7	15.4	10.5	21.4	15.2	21.2	10.3
Bahia	10.1	5.1	18.4	10.0	5.4	18.0	10.1	5.4	18.2	12.9	8.1	19.3	13.6	8.9	19.6	13.5	19.7	8.8
Ceará	13.0	7.1	21.2	14.8	9.0	22.3	13.1	7.7	20.9	13.4	8.6	19.7	14.9	10.0	20.8	14.0	20.0	9.4
Distrito Federal	13.4	7.7	21.8	14.8	9.1	22.7	15.0	9.3	22.7	12.2	7.8	18.8	13.0	8.4	19.1	12.6	18.9	8.2
Espírito Santo	11.6	6.1	20.1	12.3	7.1	20.3	12.1	6.9	20.2	11.9	7.3	18.4	12.7	8.2	18.8	12.7	18.7	8.2
Goiás	10.0	4.9	18.3	10.1	5.3	18.3	10.1	5.5	18.0	12.8	8.1	19.5	13.6	9.0	19.7	13.6	19.6	8.9
Maranhão	11.0	5.7	19.3	10.8	6.1	18.7	11.3	6.1	19.3	12.5	7.9	19.0	13.3	8.6	19.4	13.6	19.8	9.0
Mato Grosso	10.2	5.2	18.4	10.6	5.7	19.0	10.7	5.8	19.1	13.0	8.2	19.6	13.9	9.1	20.0	14.0	20.2	9.2
Mato Grosso do Sul	10.2	5.2	18.7	10.7	5.9	18.6	11.2	6.2	19.4	12.4	7.8	19.0	12.8	8.3	18.9	13.3	19.4	8.7
Minas Gerais	11.9	6.5	20.0	12.9	7.7	21.0	11.4	6.3	19.6	12.3	7.8	18.9	13.0	8.4	19.1	12.1	18.1	7.7
Pará	11.7	6.2	20.0	12.2	7.1	20.5	12.0	6.8	20.2	12.4	7.7	19.1	13.3	8.7	19.2	13.4	19.6	8.7
Paraíba	13.5	7.6	21.9	15.2	9.5	22.7	13.9	8.4	21.7	13.9	9.0	20.3	16.1	11.1	22.0	15.4	21.3	10.5
Paraná	11.0	5.8	19.2	11.8	6.7	19.7	10.4	5.7	18.4	12.3	7.6	18.9	13.7	8.9	19.5	12.4	18.6	8.0
Pernambuco	12.2	6.7	20.7	13.3	7.9	21.0	12.8	7.4	20.6	12.3	7.8	18.8	12.8	8.3	18.8	12.8	18.9	8.2
Piauí	9.8	4.8	17.9	9.2	4.8	16.8	9.2	4.9	16.8	12.8	8.0	19.3	13.6	8.9	19.7	13.5	19.7	8.9
Rio de Janeiro	13.3	7.6	21.6	14.5	8.8	22.2	13.7	8.2	21.7	13.6	8.8	20.0	15.3	10.3	21.3	14.4	20.4	9.5
Rio Grande do Norte	9.8	5.0	18.0	10.3	5.5	18.6	10.5	5.7	18.7	12.1	7.6	18.7	12.7	8.2	18.7	13.3	19.4	8.7
Rio Grande do Sul	11.4	6.1	19.7	11.1	6.4	19.1	11.3	6.4	19.3	12.1	7.6	18.7	12.6	8.0	18.6	12.6	18.9	8.1
Rondônia	13.2	7.2	21.6	13.5	8.0	21.2	14.0	8.4	21.7	13.7	8.5	20.4	15.6	10.4	21.8	15.4	21.6	10.3
Roraima	11.0	5.7	19.3	11.7	6.4	20.2	11.7	6.5	19.7	13.8	8.8	20.4	15.1	10.1	21.1	15.2	21.2	10.2
São Paulo	10.7	5.6	18.9	11.0	6.2	19.3	11.9	6.8	20.2	12.1	7.6	18.7	12.4	7.9	18.5	12.7	18.8	8.1
Santa Catarina	12.6	6.8	20.9	13.1	7.8	21.1	12.5	7.4	20.6	12.9	8.1	19.4	13.4	8.7	19.4	13.3	19.4	8.7
Sergipe	11.3	6.1	19.8	11.7	6.8	19.9	12.3	7.1	20.4	11.4	7.0	17.9	11.7	7.4	17.7	11.8	17.8	7.5
Tocantins	10.6	5.6	19.0	11.3	6.4	19.6	10.5	5.8	18.3	12.4	7.8	18.9	13.4	8.7	19.3	13.3	19.5	8.7

U.I.: uncertainty interval; * age-standardized.
