# Supplementary material for: Physical activity to prevent stroke mortality in Brazil (1990-2019)
Source: Rev Soc Bras Med Trop. 2022 Jan 28;55(Suppl 1):e0252-2021. doi: 10.1590/0037-8682-0252-2021 (PMC9020380; doi:10.1590/0037-8682-0252-2021)
Supplement: Supplementary file 6 [file 1678-9849-rsbmt-55-s01-e0252-2021-supp6.pdf]

**SUPPLEMENTARY TABLE 6**: Mortality rate (per 100,000 inhabitants) due to stroke attributable to low physical activity, and population attributable fraction in Brazilian male population aged 50-69 years in 1990, 2010, and 2019.

| | Male (aged 50-69 years) | | | | | | | | | | | | | | | | | |
|---|---|---|---|---|---|---|---|---|---|---|---|---|---|---|---|---|---|---|
| | 1990 | | | | | | 2010 | | | | | | 2019 | | | | | |
| | Rate* | 95%UI | | PAF | 95%UI | | Rate* | 95%UI | | PAF | 95%UI | | Rate* | 95%UI | | PAF | 95%UI | |
| Acre | 4.2 | 0.3 | 12.2 | 2.4 | 0.2 | 7.0 | 3.1 | 0.2 | 8.1 | 2.6 | 0.2 | 6.6 | 2.8 | 0.2 | 7.2 | 2.6 | 0.2 | 6.4 |
| Alagoas | 6.9 | 0.4 | 22.1 | 2.3 | 0.1 | 7.2 | 4.7 | 0.3 | 1.4 | 2.3 | 0.1 | 6.8 | 4.0 | 0.3 | 11.9 | 2.3 | 0.1 | 6.8 |
| Amapá | 3.3 | 0.2 | 10.1 | 2.2 | 0.1 | 6.7 | 2.5 | 0.2 | 7.4 | 2.2 | 0.2 | 6.5 | 2.4 | 0.2 | 7.2 | 2.2 | 0.2 | 6.4 |
| Amazonas | 4.5 | 0.3 | 13.8 | 2.2 | 0.1 | 6.5 | 2.3 | 0.2 | 6.5 | 2.0 | 0.1 | 5.6 | 2.2 | 0.1 | 5.9 | 2.1 | 0.1 | 5.7 |
| Bahia | 4.7 | 0.3 | 15.4 | 2.0 | 0.1 | 6.4 | 2.8 | 0.2 | 8.9 | 1.7 | 0.1 | 5.7 | 2.6 | 0.2 | 8.7 | 1.8 | 0.1 | 5.8 |
| Ceará | 4.7 | 0.3 | 13.5 | 2.5 | 0.2 | 6.9 | 3.5 | 0.3 | 9.3 | 2.8 | 0.2 | 7.0 | 2.9 | 0.2 | 7.6 | 2.4 | 0.2 | 6.2 |
| Distrito Federal | 5.0 | 0.3 | 14.2 | 2.2 | 0.1 | 6.1 | 2.4 | 0.2 | 6.2 | 2.4 | 0.2 | 6.3 | 1.9 | 0.2 | 4.8 | 2.5 | 0.2 | 6.2 |
| Espírito Santo | 7.5 | 0.5 | 22.6 | 2.2 | 0.1 | 6.5 | 3.1 | 0.2 | 8.8 | 2.1 | 0.1 | 6.0 | 2.7 | 0.2 | 7.7 | 2.2 | 0.2 | 6.1 |
| Goiás | 6.0 | 0.4 | 20.0 | 1.9 | 0.1 | 6.2 | 2.1 | 0.1 | 6.6 | 1.7 | 0.1 | 5.5 | 1.9 | 0.1 | 5.9 | 1.9 | 0.1 | 5.7 |
| Maranhão | 6.4 | 0.4 | 21.5 | 2.2 | 0.1 | 7.0 | 3.8 | 0.2 | 1.1 | 2.2 | 0.1 | 6.1 | 4.1 | 0.3 | 11.6 | 2.2 | 0.2 | 6.1 |
| Mato Grosso | 4.3 | 0.3 | 14.1 | 2.2 | 0.1 | 7.1 | 2.6 | 0.2 | 7.8 | 2.0 | 0.1 | 6.0 | 2.0 | 0.1 | 6.0 | 2.1 | 0.2 | 5.8 |
| Mato Grosso do Sul | 5.0 | 0.3 | 16.1 | 1.9 | 0.1 | 6.0 | 2.4 | 0.2 | 7.8 | 1.7 | 0.1 | 5.6 | 2.1 | 0.1 | 6.1 | 2.0 | 0.1 | 5.7 |
| Minas Gerais | 7.4 | 0.4 | 21.8 | 2.2 | 0.1 | 6.6 | 2.9 | 0.2 | 8.6 | 2.1 | 0.1 | 6.3 | 2.1 | 0.1 | 6.5 | 2.0 | 0.1 | 6.0 |
| Pará | 5.9 | 0.3 | 17.7 | 2.4 | 0.1 | 7.2 | 3.2 | 0.2 | 9.5 | 2.2 | 0.1 | 6.6 | 2.8 | 0.2 | 8.3 | 2.2 | 0.2 | 6.5 |
| Paraíba | 5.1 | 0.3 | 14.1 | 2.8 | 0.2 | 7.7 | 4.6 | 0.4 | 1.1 | 3.1 | 0.3 | 7.4 | 3.2 | 0.3 | 7.8 | 2.8 | 0.3 | 6.7 |
| Paraná | 7.9 | 0.5 | 24.2 | 2.2 | 0.1 | 6.9 | 3.2 | 0.2 | 9.6 | 2.3 | 0.2 | 6.9 | 2.5 | 0.2 | 7.8 | 2.2 | 0.2 | 6.5 |
| Pernambuco | 6.3 | 0.4 | 18.2 | 2.3 | 0.1 | 6.8 | 3.4 | 0.2 | 9.3 | 2.2 | 0.2 | 6.1 | 3.1 | 0.2 | 8.8 | 2.1 | 0.1 | 6.0 |
| Piauí | 5.0 | 0.3 | 15.8 | 1.9 | 0.1 | 6.2 | 2.4 | 0.2 | 8.0 | 1.7 | 0.1 | 5.8 | 2.0 | 0.1 | 6.4 | 1.8 | 0.1 | 5.6 |
| Rio de Janeiro | 9.9 | 0.6 | 27.2 | 2.5 | 0.2 | 6.9 | 4.4 | 0.4 | 1.1 | 2.6 | 0.2 | 6.6 | 3.4 | 0.3 | 8.8 | 2.5 | 0.2 | 6.5 |
| Rio Grande do Norte | 3.3 | 0.2 | 10.9 | 2.0 | 0.1 | 6.6 | 1.7 | 0.1 | 5.6 | 1.8 | 0.1 | 6.0 | 1.6 | 0.1 | 5.1 | 1.9 | 0.1 | 6.0 |
| Rio Grande do Sul | 6.3 | 0.4 | 19.2 | 2.3 | 0.1 | 6.9 | 3.1 | 0.2 | 8.4 | 2.4 | 0.2 | 6.5 | 2.8 | 0.2 | 7.7 | 2.5 | 0.2 | 6.8 |
| Rondônia | 7.1 | 0.5 | 20.6 | 2.7 | 0.2 | 7.6 | 3.3 | 0.3 | 8.4 | 2.8 | 0.2 | 7.1 | 3.1 | 0.3 | 7.7 | 2.9 | 0.3 | 7.3 |
| Roraima | 4.3 | 0.3 | 13.6 | 2.1 | 0.1 | 6.4 | 1.7 | 0.1 | 5.5 | 1.8 | 0.1 | 5.9 | 1.9 | 0.1 | 5.8 | 2.0 | 0.1 | 5.9 |
| São Paulo | 6.0 | 0.4 | 18.9 | 2.1 | 0.1 | 6.7 | 2.6 | 0.2 | 7.8 | 2.0 | 0.1 | 6.0 | 2.3 | 0.2 | 6.4 | 2.3 | 0.2 | 6.5 |
| Santa Catarina | 7.0 | 0.4 | 20.2 | 2.4 | 0.1 | 7.0 | 2.5 | 0.2 | 6.7 | 2.5 | 0.2 | 6.6 | 2.1 | 0.2 | 5.7 | 2.6 | 0.2 | 6.8 |
| Sergipe | 5.9 | 0.4 | 17.3 | 2.4 | 0.2 | 7.1 | 3.0 | 0.2 | 8.7 | 2.2 | 0.2 | 6.3 | 2.9 | 0.2 | 8.1 | 2.4 | 0.2 | 6.4 |
| Tocantins | 4.3 | 0.3 | 14.0 | 2.1 | 0.1 | 6.6 | 2.9 | 0.2 | 8.9 | 2.2 | 0.1 | 6.5 | 2.8 | 0.2 | 8.3 | 2.2 | 0.1 | 6.5 |

PAF: population attributable fraction; UI: uncertainty interval; *Rate per 100,000 inhabitant.
