# Supplementary material for: Physical activity to prevent stroke mortality in Brazil (1990-2019)
Source: Rev Soc Bras Med Trop. 2022 Jan 28;55(Suppl 1):e0252-2021. doi: 10.1590/0037-8682-0252-2021 (PMC9020380; doi:10.1590/0037-8682-0252-2021)
Supplement: Supplementary file 7 [file 1678-9849-rsbmt-55-s01-e0252-2021-supp7.pdf]

SUPPLEMENTARY TABLE 7: Mortality rate (per 100,000 inhabitants) due to stroke attributable to low physical activity, and population attributable fraction in Brazilian male population aged ≥ 70 years in 1990, 2010, and 2019.

	Male (aged 70+ years)																	
	1990						2010						2019					
	Rate*	95%UI		PAF	95%UI		Rate*	95%UI		PAF	95%UI		Rate*	95%UI		PAF	95%UI	
Acre	66.5	10.2	141.8	6.3	1.0	13.4	69.2	13.8	128.5	8.0	1.6	14.8	65.6	13.3	123.1	8.3	1.7	15.2
Alagoas	96.0	11.3	215.8	5.8	0.7	13.1	81.9	13.9	167.4	7.4	1.3	14.7	74.2	13.8	153.9	7.4	1.3	14.7
Amapá	66.0	9.2	141.6	6.1	0.9	13.2	51.7	10.1	101.7	7.2	1.3	13.9	54.6	10.4	104.6	7.7	1.5	14.6
Amazonas	60.9	8.1	134.1	5.6	0.8	12.0	61.0	12.9	114.6	7.9	1.7	14.5	59.7	12.9	113.4	8.2	1.8	15.0
Bahia	63.4	7.5	147.3	5.6	0.6	12.5	73.5	12.7	152.0	7.5	1.2	14.8	62.4	10.0	126.6	7.3	1.2	14.4
Ceará	79.3	11.1	169.0	6.9	1.0	14.4	86.7	20.4	161.7	9.1	2.0	16.5	76.6	16.7	143.0	8.7	1.9	16.1
Distrito Federal	63.0	9.0	137.3	5.1	0.7	11.1	68.7	13.4	129.9	7.9	1.6	14.9	55.6	12.7	106.4	8.3	1.8	15.3
Espírito Santo	95.6	11.4	211.6	5.9	0.7	12.8	75.7	16.1	148.5	7.8	1.6	15.0	61.7	11.3	121.2	7.8	1.4	14.9
Goiás	65.9	7.3	157.2	4.9	0.6	11.4	44.1	7.2	91.5	6.3	1.0	13.0	41.1	6.7	85.8	6.7	1.1	13.7
Maranhão	83.9	10.3	191.8	5.9	0.8	13.3	96.5	19.1	186.9	8.8	1.8	16.4	10.8	19.5	209.9	8.6	1.6	15.9
Mato Grosso	62.4	7.4	144.4	5.3	0.6	12.1	46.9	71.7	100.3	6.4	1.0	13.4	37.9	5.4	81.3	6.7	1.0	13.8
Mato Grosso do Sul	62.7	7.8	148.2	4.9	0.6	11.3	54.7	9.2	111.7	6.9	1.2	13.8	46.2	7.8	93.0	7.3	1.2	14.4
Minas Gerais	73.7	9.8	161.2	5.6	0.7	12.1	55.8	11.1	106.9	7.7	1.5	14.6	46.8	9.4	88.4	8.0	1.7	14.8
Pará	83.9	10.8	189.0	5.7	0.8	12.2	76.6	17.5	143.7	8.4	1.7	15.4	72.7	14.5	141.6	8.5	1.7	15.8
Paraíba	74.1	10.7	154.5	7.1	1.0	14.8	89.1	21.0	162.7	9.5	2.2	16.8	66.1	15.0	121.3	9.1	2.1	16.1
Paraná	94.7	10.9	217.4	5.4	0.6	12.2	67.4	11.1	136.5	7.0	1.1	14.1	58.3	8.5	121.3	7.2	1.1	14.6
Pernambuco	84.0	12.0	179.5	6.0	0.8	12.8	70.9	14.1	140.8	7.6	1.5	14.9	65.1	13.0	129.0	7.5	1.4	14.4
Piauí	87.7	11.0	205.3	5.6	0.7	13.1	78.2	13.0	157.1	7.7	1.3	15.4	69.1	11.7	138.1	8.0	1.4	15.6
Rio de Janeiro	92.3	13.9	194.3	6.0	0.9	12.5	66.2	13.4	125.1	7.7	1.5	14.4	53.2	11.3	97.8	7.9	1.7	14.4
Rio Grande do Norte	60.7	7.2	137.9	5.8	0.7	13.0	48.7	7.0	103.0	7.3	1.1	15.2	41.5	6.5	88.5	7.1	1.2	14.6
Rio Grande do Sul	76.7	9.5	169.5	5.6	0.7	12.4	64.2	11.7	125.9	7.4	1.3	14.4	54.2	9.8	105.8	7.6	1.4	14.9
Rondônia	63.1	7.4	143.2	4.5	0.5	10.1	64.8	14.9	116.0	8.6	2.0	15.3	66.5	15.8	123.0	9.2	2.2	16.3
Roraima	63.3	8.1	147.8	4.9	0.6	11.2	44.1	7.7	87.3	7.1	1.3	13.8	46.2	7.9	88.8	7.5	1.3	14.6
São Paulo	67.6	8.5	157.4	5.3	0.7	12.0	53.7	9.9	108.3	7.1	1.3	14.0	42.7	7.3	84.9	7.4	1.3	14.2
Santa Catarina	95.3	12.9	207.4	5.8	0.7	12.7	65.6	12.7	123.6	8.1	1.6	15.1	52.6	11.6	99.1	8.3	1.9	15.2
Sergipe	82.9	9.9	182.7	5.7	0.7	12.7	73.1	13.1	148.3	8.0	1.5	15.2	65.6	12.9	125.6	8.0	1.5	15.3
Tocantins	63.2	7.7	148.3	5.3	0.6	12.0	66.4	13.3	129.9	7.9	1.6	15.3	75.4	14.3	147.6	8.0	1.5	15.6

PAF: population attributable fraction; UI: uncertainty interval; *Rate per 100,000 inhabitant.
