# Supplementary material for: Physical activity to prevent stroke mortality in Brazil (1990-2019)
Source: Rev Soc Bras Med Trop. 2022 Jan 28;55(Suppl 1):e0252-2021. doi: 10.1590/0037-8682-0252-2021 (PMC9020380; doi:10.1590/0037-8682-0252-2021)
Supplement: Supplementary file 8 [file 1678-9849-rsbmt-55-s01-e0252-2021-supp8.pdf]

SUPPLEMENTARY TABLE 8: Mortality rate (per 100,000 inhabitants) due to stroke attributable to low physical activity, and population attributable fraction in Brazilian female population aged 25-49 years in 1990, 2010, and 2019.

	Female (aged 25-49 years)																	
	1990						2010						2019					
	Rate*	95%UI		PAF	95%UI		Rate*	95%UI		PAF	95%UI		Rate*	95%UI		PAF	95%UI	
Acre	0.2	0.0	0.6	1.1	0.1	3.1	0.1	0.0	0.3	1.0	0.1	2.7	0.1	0.0	0.2	1.0	0.1	2.6
Alagoas	0.4	0.0	1.1	1.4	0.1	3.9	0.2	0.0	0.6	1.2	0.1	3.1	0.2	0.0	0.5	1.2	0.1	3.2
Amapá	0.2	0.0	0.5	1.3	0.1	3.6	0.1	0.0	0.2	1.1	0.1	2.9	0.1	0.0	0.3	1.1	0.1	2.8
Amazonas	0.2	0.0	0.6	1.5	0.1	3.8	0.1	0.0	0.3	1.0	0.1	2.7	0.1	0.0	0.2	1.0	0.1	2.7
Bahia	0.3	0.0	0.9	1.2	0.1	3.3	0.1	0.0	0.4	0.9	0.1	2.3	0.1	0.0	0.3	0.8	0.0	2.3
Ceará	0.2	0.0	0.6	1.0	0.1	2.9	0.1	0.0	0.3	1.0	0.1	2.5	0.1	0.0	0.3	0.9	0.0	2.4
Distrito Federal	0.2	0.0	0.7	1.0	0.0	2.8	0.1	0.0	0.2	0.7	0.0	2.0	0.0	0.0	0.1	0.7	0.0	2.0
Espírito Santo	0.3	0.0	0.9	1.0	0.0	3.1	0.1	0.0	0.4	0.9	0.0	2.5	0.1	0.0	0.3	0.8	0.0	2.5
Goiás	0.4	0.0	1.1	1.1	0.1	3.1	0.1	0.0	0.3	0.8	0.0	2.4	0.1	0.0	0.2	0.9	0.0	2.4
Maranhão	0.2	0.0	0.7	1.1	0.1	3.2	0.2	0.0	0.4	0.9	0.0	2.6	0.1	0.0	0.4	0.9	0.0	2.6
Mato Grosso	0.2	0.0	0.7	1.2	0.1	3.3	0.1	0.0	0.3	0.9	0.0	2.4	0.1	0.0	0.2	0.9	0.0	2.4
Mato Grosso do Sul	0.3	0.0	0.8	1.0	0.0	2.8	0.1	0.0	0.3	0.8	0.0	2.4	0.1	0.0	0.2	0.8	0.0	2.3
Minas Gerais	0.4	0.0	1.1	1.1	0.1	3.2	0.1	0.0	0.3	0.8	0.0	2.3	0.1	0.0	0.2	0.7	0.0	2.1
Pará	0.3	0.0	0.9	1.5	0.1	4.3	0.1	0.0	0.4	1.1	0.1	3.0	0.1	0.0	0.3	1.0	0.0	3.0
Paraíba	0.3	0.0	0.8	1.2	0.1	3.2	0.2	0.0	0.5	1.2	0.1	2.8	0.1	0.0	0.4	1.1	0.1	2.6
Paraná	0.3	0.0	1.0	1.3	0.1	3.8	0.1	0.0	0.4	1.2	0.1	3.2	0.1	0.0	0.3	1.0	0.0	3.0
Pernambuco	0.3	0.0	0.8	1.2	0.1	3.5	0.1	0.0	0.4	0.9	0.0	2.5	0.1	0.0	0.3	0.8	0.0	2.2
Piauí	0.3	0.0	0.8	1.1	0.0	3.3	0.1	0.0	0.4	1.0	0.1	2.7	0.1	0.0	0.3	1.0	0.1	2.7
Rio de Janeiro	0.4	0.0	1.1	1.1	0.1	3.0	0.1	0.0	0.4	0.9	0.1	2.3	0.1	0.0	0.3	0.8	0.0	2.2
Rio Grande do Norte	0.2	0.0	0.6	1.1	0.0	3.4	0.1	0.0	0.3	0.9	0.0	2.7	0.1	0.0	0.2	1.0	0.0	2.8
Rio Grande do Sul	0.3	0.0	0.9	1.2	0.1	3.6	0.1	0.0	0.3	0.9	0.0	2.7	0.1	0.0	0.2	0.9	0.0	2.7
Rondônia	0.3	0.0	0.8	1.4	0.1	3.7	0.1	0.0	0.4	1.2	0.1	3.1	0.1	0.0	0.3	1.2	0.1	3.0
Roraima	0.2	0.0	0.6	1.4	0.1	3.6	0.1	0.0	0.2	1.1	0.1	2.8	0.1	0.0	0.2	1.1	0.1	2.9
São Paulo	0.3	0.0	0.8	1.1	0.1	3.3	0.1	0.0	0.3	0.8	0.0	2.5	0.1	0.0	0.2	0.9	0.0	2.5
Santa Catarina	0.3	0.0	0.7	1.2	0.1	3.4	0.1	0.0	0.2	1.0	0.0	2.7	0.1	0.0	0.2	0.9	0.0	2.7
Sergipe	0.3	0.0	0.8	1.2	0.0	3.7	0.1	0.0	0.4	0.9	0.0	2.7	0.1	0.0	0.3	0.8	0.0	2.6
Tocantins	0.2	0.0	0.7	0.9	0.0	2.8	0.1	0.0	0.4	0.9	0.0	2.6	0.1	0.0	0.3	0.9	0.0	2.6

PAF: population attributable fraction; UI: uncertainty interval; *Rate per 100,000 inhabitant.
