# Supplementary material for: Physical activity to prevent stroke mortality in Brazil (1990-2019)
Source: Rev Soc Bras Med Trop. 2022 Jan 28;55(Suppl 1):e0252-2021. doi: 10.1590/0037-8682-0252-2021 (PMC9020380; doi:10.1590/0037-8682-0252-2021)
Supplement: Supplementary file 9 [file 1678-9849-rsbmt-55-s01-e0252-2021-supp9.pdf]

**SUPPLEMENTARY TABLE 9**: Mortality rate (per 100,000 inhabitants) due to stroke attributable to low physical activity, and population attributable fraction in Brazilian female population aged 50-69 years in 1990, 2010, and 2019.

| | Female (aged 50-69 years) | | | | | | | | | | | | | | | | | |
|---|---|---|---|---|---|---|---|---|---|---|---|---|---|---|---|---|---|---|
| | 1990 | | | | | | 2010 | | | | | | 2019 | | | | | |
| | Rate* | 95%UI | | PAF | 95%UI | | Rate* | 95%UI | | PAF | 95%UI | | Rate* | 95%UI | | PAF | 95%UI | |
| Acre | 2.7 | 0.3 | 6.8 | 2.0 | 0.2 | 4.9 | 2.0 | 0.3 | 4.5 | 2.0 | 0.3 | 4.6 | 1.6 | 0.2 | 3.6 | 2.1 | 0.3 | 4.6 |
| Alagoas | 4.9 | 0.5 | 12.3 | 2.2 | 0.2 | 5.5 | 3.1 | 0.4 | 7.1 | 2.2 | 0.3 | 5.0 | 2.8 | 0.4 | 6.5 | 2.3 | 0.3 | 5.1 |
| Amapá | 2.4 | 0.3 | 5.8 | 2.2 | 0.2 | 5.2 | 1.5 | 0.2 | 3.6 | 2.2 | 0.3 | 5.0 | 1.8 | 0.2 | 4.1 | 2.3 | 0.3 | 5.2 |
| Amazonas | 3.5 | 0.4 | 8.4 | 2.2 | 0.3 | 5.2 | 1.5 | 0.3 | 3.2 | 2.0 | 0.3 | 4.1 | 1.4 | 0.2 | 2.9 | 2.1 | 0.4 | 4.3 |
| Bahia | 3.8 | 0.4 | 9.3 | 2.1 | 0.2 | 5.1 | 1.9 | 0.2 | 4.5 | 1.8 | 0.2 | 4.2 | 1.5 | 0.2 | 3.7 | 1.8 | 0.2 | 4.1 |
| Ceará | 2.3 | 0.3 | 5.8 | 1.9 | 0.2 | 4.6 | 1.7 | 0.2 | 3.9 | 1.9 | 0.2 | 4.4 | 1.4 | 0.2 | 3.4 | 1.9 | 0.2 | 4.3 |
| Distrito Federal | 3.1 | 0.3 | 8.2 | 1.8 | 0.2 | 4.6 | 1.0 | 0.1 | 2.4 | 1.4 | 0.2 | 3.5 | 0.8 | 0.1 | 2.0 | 1.6 | 0.2 | 3.9 |
| Espírito Santo | 4.0 | 0.4 | 10.5 | 1.9 | 0.2 | 4.9 | 1.5 | 0.2 | 3.6 | 1.7 | 0.2 | 4.1 | 1.4 | 0.2 | 3.4 | 1.8 | 0.2 | 4.3 |
| Goiás | 4.6 | 0.5 | 11.9 | 2.0 | 0.2 | 5.0 | 1.5 | 0.2 | 3.4 | 1.7 | 0.2 | 3.9 | 1.3 | 0.2 | 3.3 | 1.8 | 0.2 | 4.2 |
| Maranhão | 2.2 | 0.2 | 6.1 | 1.7 | 0.2 | 4.5 | 2.0 | 0.2 | 5.0 | 1.7 | 0.2 | 4.0 | 1.9 | 0.2 | 4.6 | 1.8 | 0.2 | 4.1 |
| Mato Grosso | 3.3 | 0.3 | 8.5 | 2.1 | 0.2 | 5.3 | 1.8 | 0.3 | 3.9 | 2.0 | 0.3 | 4.3 | 1.4 | 0.2 | 3.1 | 2.2 | 0.4 | 4.6 |
| Mato Grosso do Sul | 3.6 | 0.4 | 9.2 | 1.8 | 0.2 | 4.6 | 1.6 | 0.2 | 3.8 | 1.7 | 0.2 | 4.1 | 1.3 | 0.2 | 2.9 | 1.9 | 0.3 | 4.1 |
| Minas Gerais | 4.3 | 0.4 | 10.6 | 2.1 | 0.2 | 5.3 | 1.5 | 0.2 | 3.6 | 1.8 | 0.2 | 4.3 | 1.2 | 0.1 | 2.8 | 1.7 | 0.2 | 4.0 |
| Pará | 4.0 | 0.4 | 10.5 | 2.2 | 0.2 | 5.7 | 2.1 | 0.3 | 5.1 | 2.1 | 0.2 | 4.8 | 1.9 | 0.3 | 4.3 | 2.3 | 0.3 | 5.1 |
| Paraíba | 3.1 | 0.4 | 7.6 | 2.2 | 0.3 | 5.3 | 2.1 | 0.3 | 4.6 | 2.2 | 0.3 | 4.8 | 1.6 | 0.2 | 3.6 | 2.1 | 0.3 | 4.5 |
| Paraná | 4.9 | 0.5 | 12.6 | 2.2 | 0.2 | 5.6 | 1.7 | 0.2 | 4.2 | 2.0 | 0.2 | 4.9 | 1.4 | 0.2 | 3.5 | 2.0 | 0.2 | 4.8 |
| Pernambuco | 4.4 | 0.5 | 11.2 | 2.1 | 0.2 | 5.3 | 1.6 | 0.2 | 3.9 | 1.7 | 0.2 | 4.0 | 1.7 | 0.2 | 4.1 | 1.7 | 0.2 | 3.9 |
| Piauí | 2.9 | 0.3 | 7.2 | 1.9 | 0.2 | 4.6 | 2.1 | 0.3 | 4.5 | 1.8 | 0.2 | 4.3 | 1.5 | 0.2 | 3.5 | 1.9 | 0.2 | 4.3 |
| Rio de Janeiro | 5.6 | 0.6 | 13.1 | 2.2 | 0.3 | 5.3 | 1.1 | 0.1 | 2.8 | 2.0 | 0.3 | 4.3 | 1.5 | 0.2 | 3.7 | 2.0 | 0.3 | 4.4 |
| Rio Grande do Norte | 2.3 | 0.2 | 6.1 | 2.0 | 0.2 | 5.3 | 1.4 | 0.1 | 3.6 | 1.8 | 0.2 | 4.4 | 1.7 | 0.2 | 3.7 | 2.1 | 0.3 | 4.8 |
| Rio Grande do Sul | 4.0 | 0.4 | 10.4 | 2.3 | 0.2 | 5.9 | 2.1 | 0.3 | 4.5 | 1.7 | 0.2 | 4.3 | 1.1 | 0.2 | 2.7 | 1.9 | 0.2 | 4.6 |
| Rondônia | 4.6 | 0.5 | 11.2 | 2.2 | 0.2 | 5.3 | 1.8 | 0.3 | 3.6 | 2.2 | 0.3 | 4.8 | 1.4 | 0.2 | 3.2 | 2.3 | 0.3 | 5.1 |
| Roraima | 3.5 | 0.4 | 8.3 | 2.3 | 0.2 | 5.4 | 1.2 | 0.1 | 3.2 | 2.6 | 0.4 | 5.4 | 1.7 | 0.3 | 3.8 | 2.9 | 0.5 | 5.7 |
| São Paulo | 3.6 | 0.4 | 9.2 | 2.1 | 0.2 | 5.5 | 1.1 | 0.1 | 2.7 | 1.6 | 0.2 | 4.2 | 1.8 | 0.3 | 3.7 | 1.9 | 0.2 | 4.5 |
| Santa Catarina | 4.2 | 0.5 | 10.6 | 2.2 | 0.2 | 5.5 | 1.6 | 0.2 | 4.2 | 1.7 | 0.2 | 4.2 | 1.1 | 0.1 | 2.8 | 1.9 | 0.2 | 4.5 |
| Sergipe | 3.2 | 0.3 | 8.5 | 1.9 | 0.2 | 5.2 | 1.8 | 0.2 | 4.3 | 1.7 | 0.2 | 4.3 | 1.0 | 0.1 | 2.5 | 1.8 | 0.2 | 4.4 |
| Tocantins | 2.6 | 0.3 | 6.6 | 1.7 | 0.2 | 4.2 | 1.7 | 0.2 | 4.1 | 1.9 | 0.2 | 4.4 | 1.6 | 0.2 | 3.7 | 2.1 | 0.3 | 4.7 |

PAF: population attributable fraction; UI: uncertainty interval; *Rate per 100,000 inhabitant.
