# Supplementary material for: Physical activity to prevent stroke mortality in Brazil (1990-2019)
Source: Rev Soc Bras Med Trop. 2022 Jan 28;55(Suppl 1):e0252-2021. doi: 10.1590/0037-8682-0252-2021 (PMC9020380; doi:10.1590/0037-8682-0252-2021)
Supplement: Supplementary file 10 [file 1678-9849-rsbmt-55-s01-e0252-2021-supp10.pdf]

SUPPLEMENTARY TABLE 10: Mortality rate (per 100,000 inhabitants) due to stroke attributable to low physical activity, and population attributable fraction in Brazilian female population aged ≥ 70 years in 1990, 2010, and 2019.

	Female (aged 70+ years)																	
	1990						2010						2019					
	Rate*	95%UI		PAF	95%UI		Rate*	95%UI		PAF	95%UI		Rate*	95%UI		PAF	95%UI	
Acre	70.4	15.9	131.8	7.7	1.6	14.3	60.9	17.4	105.0	9.7	3.0	16.7	59.6	16.6	104.7	9.7	2.7	16.6
Alagoas	118.9	25.5	230.2	8.2	1.8	15.5	95.3	27.8	167.0	10.3	3.1	17.4	89.6	25.7	159.5	10.3	3.1	17.5
Amapá	68.3	13.8	128.5	8.5	1.9	15.8	54.8	15.6	97.7	9.5	2.8	16.5	56.0	15.7	101.7	9.9	2.8	16.9
Amazonas	94.9	21.8	175.6	7.9	1.7	14.4	68.7	22.5	116.0	10.3	3.5	16.9	65.3	21.3	114.5	10.6	3.4	17.8
Bahia	92.6	19.9	177.0	8.0	1.7	15.0	71.7	20.0	126.6	10.4	3.1	17.7	65.0	18.2	117.0	10.5	3.0	17.8
Ceará	82.8	18.5	159.5	8.4	1.9	15.7	84.9	25.4	149.1	10.6	3.3	18.0	75.7	21.1	136.3	10.4	3.0	17.6
Distrito Federal	68.4	12.6	135.2	6.3	1.1	12.3	58.4	15.2	107.6	8.8	2.2	15.7	48.1	11.5	87.2	9.0	2.2	16.0
Espírito Santo	101.5	20.4	199.5	7.5	1.5	14.5	69.2	17.0	126.0	9.1	2.3	16.3	58.2	13.7	108.8	9.0	2.2	16.4
Goiás	92.4	19.7	182.4	7.1	1.5	13.7	55.6	15.6	98.1	9.0	2.6	15.6	51.4	14.0	92.5	9.5	2.7	16.4
Maranhão	57.7	11.3	116.7	8.1	1.7	15.4	95.7	26.4	170.3	10.3	3.0	17.8	87.6	23.4	159.5	10.1	2.8	17.5
Mato Grosso	71.9	14.8	139.6	7.5	1.6	14.1	60.5	17.6	104.9	9.7	2.8	16.6	51.6	15.2	89.8	10.0	3.2	16.9
Mato Grosso do Sul	73.9	14.6	144.0	6.8	1.4	13.1	58.1	15.5	103.0	9.1	2.5	15.8	50.4	13.6	92.5	9.1	2.4	16.0
Minas Gerais	82.7	15.3	159.8	7.4	1.4	14.1	60.8	16.4	106.1	9.5	2.6	16.6	50.0	13.4	89.6	9.6	2.5	16.6
Pará	113.0	21.8	218.3	7.8	1.6	15.0	77.2	22.6	136.9	10.2	3.0	17.4	68.3	19.3	120.6	10.3	2.9	17.5
Paraíba	92.4	19.3	177.4	8.8	1.9	16.2	86.0	28.1	146.5	11.0	3.6	18.7	69.6	22.0	123.1	10.6	3.2	17.9
Paraná	103.5	21.3	203.7	7.1	1.4	13.8	70.8	18.6	124.7	9.4	2.5	16.6	61.7	15.8	111.0	9.6	2.4	17.0
Pernambuco	98.9	20.0	190.6	7.5	1.5	14.4	73.4	21.2	129.9	9.5	2.8	16.6	64.4	17.3	116.6	9.4	2.6	16.2
Piauí	93.5	18.9	183.6	7.8	1.7	14.8	86.8	26.0	151.1	10.7	3.3	18.0	81.4	23.0	141.8	10.7	3.0	18.3
Rio de Janeiro	107.8	23.7	200.2	8.0	1.8	14.9	66.0	20.2	114.4	9.5	2.9	16.4	53.7	15.7	95.0	9.7	2.8	16.4
Rio Grande do Norte	69.0	14.1	132.4	7.8	1.6	14.7	54.8	15.6	97.9	10.2	2.9	17.5	47.6	13.6	84.2	9.9	2.8	17.0
Rio Grande do Sul	102.0	20.3	193.4	7.8	1.5	14.8	80.1	23.1	141.1	10.1	2.9	17.4	68.9	18.4	124.0	9.9	2.8	17.4
Rondônia	77.0	14.2	149.8	5.8	1.1	11.4	56.1	15.5	98.3	9.1	2.5	15.8	59.7	16.1	108.3	9.7	2.6	16.7
Roraima	70.3	15.3	131.9	7.4	1.7	13.8	47.7	13.4	80.6	9.7	2.9	16.2	54.3	16.1	92.7	10.2	3.0	17.3
São Paulo	77.0	16.6	150.0	7.3	1.5	14.1	57.7	16.8	100.8	9.6	2.8	16.7	47.1	12.9	84.4	9.6	2.7	16.5
Santa Catarina	112.4	23.3	217.3	7.5	1.5	14.4	70.2	20.3	121.2	10.3	2.9	17.7	58.8	16.5	104.6	10.2	2.9	17.6
Sergipe	95.1	18.4	187.8	7.1	1.4	14.1	70.0	18.1	128.7	9.2	2.3	16.5	65.2	15.9	117.2	9.0	2.2	16.5
Tocantins	72.0	15.0	141.0	7.0	1.5	13.6	60.9	15.8	108.3	9.2	2.4	16.3	63.0	16.8	118.1	9.4	2.3	16.5

PAF: population attributable fraction; UI: uncertainty interval; *Rate per 100,000 inhabitant.
